# A commentary on “Eucalyptus obliqua seedling growth in organic vs. mineral soil horizons”

**DOI:** 10.3389/fpls.2015.00346

**Published:** 2015-05-18

**Authors:** Mark G. Neyland, Simon J. Grove

**Affiliations:** ^1^Forestry TasmaniaHobart, TAS, Australia; ^2^Tasmanian Museum and Art GalleryRosny, TAS, Australia

**Keywords:** eucalyptus, regeneration, Australia, burning, harvesting

In a recent paper in this journal, Barry et al. (2015) reported on a pot-based trial comparing the growth of transplanted *Eucalyptus obliqua* seedlings in mineral vs. organic soils. Unfortunately in “Forestry Management Implications” (p. 11) they make claims regarding the appropriateness of fire as a regeneration tool in temperate Australian native wet eucalypt forests following harvesting. In doing so, they ignore and/or misinterpret the work of other researchers in this field. The authors' remarks in this regard are speculative and in no way supported by their experiment, since their experimental design was pot-based rather than field-based and could not therefore incorporate either fire or forest harvesting. We also note that their experiment was based on transplanted seedlings whereas both nature and forest managers regenerate such forests from seed.

The section opens with the statement that “our results strongly question the notion prevalent in forest management (Neyland et al., 2009) that because of requisite fertility improvement of exposed mineral soil a burnt seedbed—an ashbed—is necessary to ensure adequate stocking of *E. obliqua* seedlings after timber harvest.” They then “suggest that much of fire's ashbed effect on mineral soil may be a ‘remedy’ for a problem created by fire, i.e., loss of the relatively fertile organic soil layer.” Given that the authors did not study regeneration burns, so did not measure the loss of the organic soil layer during these burns, this statement must be considered a suggestion, and nothing more. Neyland et al. (2009) mentioned neither fertility nor fertility improvement as a requirement for adequate stocking after timber harvest. Nor did Neyland et al. (2009) state that the ashbed effect is necessary. Rather, Neyland et al. (2009) defined a range of seedbed types resulting from the typically wide range of burning intensities experienced in a high-intensity regeneration burn. Their empirical study, and that of others before them (e.g., Pryor, 1960; Chambers and Attiwill, 1994; Lockett, 1998; Van der Meer et al., 1999; Bauhus et al., 2002; Van der Meer and Dignan, 2007), allowed them to point to the fundamental importance of burnt soil as part of the regeneration cycle, particularly with respect to *E. obliqua* seedling establishment, early seedling density, and early seedling growth; it also highlighted that, under field conditions, *E. obliqua* seedlings generally fail to establish on unburnt litter.

Barry et al. (2015) then cite the studies of Neyland et al. (2009) and Scott et al. (2012) to attempt to raise doubts as to the necessity of post-harvest regeneration burning for satisfactory regeneration. They state that the dispersed retention and aggregated retention silvicultural treatments studied by these authors demonstrated satisfactory regeneration despite poor burns. In doing so, Barry et al. (2015) have confounded *stocking* (the percentage of plots stocked with seedlings) with *seedling density* (the number of seedlings established per hectare). In both examples cited, the stocking was indeed in the acceptable range, but the overall seedling density was well below the levels considered necessary for future development of a productive regrowth eucalypt forest, as discussed at length in the papers cited.

Barry et al. (2015) then suggest that perhaps mechanically removing the CWD and the competing vegetation regrowth by “several bouts of repeated re-clearing prior to eucalypt re-sowing would be an appropriate site preparation technique as an alternative to burning.” This suggestion, if implemented, would not only be a severe economic impost to forest managers, it would also profoundly alter the forest's ecology, setting it on a trajectory far removed from the natural disturbance regimes to which most species in the forest are presumably adapted (Whelan, 1995; Gill et al., 2002; Bowman and Murphy, 2010; Tng et al., 2012; New, 2014). Their thinking may be based on a fallacious assumption that regeneration burns largely eliminate CWD. In fact, regeneration burns primarily combust litter and fine woody debris, with the percentage combusted decreasing roughly in proportion to piece diameter (Slijepcevic, 2001). Retained CWD can benefit eucalypt seedling establishment through maintaining more-humid microclimates and in providing some protection against browsing animals (Bailey et al., 2012). To suggest that removing the CWD after harvest might be an appropriate alternative to burning ignores a large body of research pointing to the fundamental ecological importance of coarse woody debris in forest ecosystems (Harmon et al., 1986; Spies et al., 1988; Grove, 2002). Indeed, CWD may be the primary habitat of the largest proportion of species living in a forest—particularly insects, fungi, and microorganisms (Stokland et al., 2012). For these reasons, in parts of the world where removal of CWD is entrenched in forest management (e.g., for industrial fuelwood harvesting), many dependent species can become threatened with extinction (Grove and Meggs, 2003; Ranius and Roberge, 2011; Bouget et al., 2012).

To further suggest that “repeated re-clearing” might be an appropriate alternative to burning also ignores a large body of research pointing to the detrimental effects of mechanical disturbance and to the ecologically beneficial effects of burning. For instance, Hindrum et al. (2012) demonstrated that many of the dominant wet eucalypt forest understorey plant species are most abundant following burning, while mechanical disturbance destroys root-stocks of “legacy” plants and instead promotes the regeneration of dense stands of short-lived early-successional species. Many other authors (Hickey, 1994; Ough, 2001; Harris, 2004; Neyland and Jarman, 2011) have recognized the important roles of burning and the minimisation of mechanical disturbance as part of ensuring the regeneration of the full suite of understorey plant species that were present before harvest. Deliberate additional mechanical disturbance would also have significant detrimental impacts on the soil (Rab, 1996, 2004).

To summarize our concerns, we feel that, while the main focus of Barry et al. (2015) is not contentious (to us at least), they ventured unnecessarily into areas not supported by their experimental design. In doing so, they promoted an ecologically detrimental vision of eucalypt forest management that is not consistent with the findings of evidence-based research—either theirs or that of the many researchers whose work they chose either to ignore or to misinterpret.

## Conflict of interest statement

The authors declare that the research was conducted in the absence of any commercial or financial relationships that could be construed as a potential conflict of interest.
